# Relationship between Prolactin Plasma Levels and White Matter Volume in Women with Multiple Sclerosis

**DOI:** 10.1155/2015/732539

**Published:** 2015-07-08

**Authors:** L. De Giglio, F. Marinelli, L. Prosperini, G. M. Contessa, F. Gurreri, M. C. Piattella, F. De Angelis, V. T. Barletta, V. Tomassini, P. Pantano, C. Pozzilli

**Affiliations:** ^1^Department of Neurology and Psychiatry, Sapienza University of Rome, 00185 Rome, Italy; ^2^Sant'Andrea Hospital, 00189 Rome, Italy; ^3^Italian National Agency for New Technologies, Energy and Sustainable Economic Development (ENEA), 00196 Rome, Italy; ^4^Institute of Psychological Medicine and Clinical Neurosciences, Cardiff University School of Medicine, Cardiff CF14 4XN, UK; ^5^IRCCS Fondazione Santa Lucia, 00142 Rome, Italy; ^6^IRCCS Neuromed, Pozzilli, 86077 Isernia, Italy

## Abstract

*Background*. The role of prolactin (PRL) on tissue injury and repair mechanisms in multiple sclerosis (MS) remains unclear. The aim of this work was to investigate the relationship between PRL plasma levels and brain damage as measured by magnetic resonance imaging (MRI). *Methods*. We employed a chemiluminescence immunoassay for measuring plasma levels of PRL. We used a 1.5 T scanner to acquire images and Jim 4.0 and SIENAX software to analyse them. *Results*. We included 106 women with relapsing remitting (RR) MS and stable disease in the last two months. There was no difference in PRL plasma levels between patients with and without gadolinium enhancement on MRI. PRL plasma levels correlated with white matter volume (WMV) (rho = 0.284, *p* = 0.014) but not with grey matter volume (GMV). Moreover, PRL levels predicted changes in WMV (Beta: 984, *p* = 0.034). *Conclusions*. Our data of a positive association between PRL serum levels and WMV support the role of PRL in promoting myelin repair as documented in animal models of demyelination. The lack of an increase of PRL in the presence of gadolinium enhancement, contrasts with the view considering this hormone as an immune-stimulating and detrimental factor in the inflammatory process associated with MS.

## 1. Background

In addition to its primary roles in mammary gland development, as well as the initiation and maintenance of lactation, prolactin (PRL) is believed to be involved in a number of physiological roles, including immune system modulation promoting B cell autoreactivity and cell proliferation promoting myelin repair [1, 2].

However, the role of PRL in tissue injury and repair mechanisms related to multiple sclerosis (MS) is still debated. Prolactin (PRL) has long been proposed as an immune-stimulating and detrimental factor in autoimmune disorders. However, recent findings have challenged this common view, showing that PRL does not play a crucial role in the development of experimental autoimmune encephalomyelitis, the animal model for multiple sclerosis (MS), and even protects against adjuvant induced model of rheumatoid arthritis [3, 4]. Some authors reported elevated PRL level among female MS patients compared with controls and patients with Clinically Isolated Syndrome (CIS) [5] while some others failed to demonstrate this association [6]. A relationship between PRL and MS relapses has been also proposed: higher serum levels of PRL were found in patients with RRMS during relapse and in patients with optic neuritis when compared to healthy controls [7, 8]. Furthermore, there is evidence that a reduction in number of gadolinium- (Gd-) enhancing lesions by a single intravenous infusion of methylprednisolone is correlated with a parallel decline of PRL [9].

Recently, experimental evidences suggest a role of PRL in endogenous repair of white matter damage. PRL signalling is both necessary and sufficient for the pregnancy-induced increase in oligodendrocyte precursor cells proliferation, and, most strikingly, PRL treatments mimic the regenerative effects of pregnancy and promote white matter repair and remyelination [2].

The aim of the present study is to investigate the role of PRL on damage and repair in MS; with this objective we analysed the relationship between PRL plasma levels and brain MRI data in a cohort of female patients.

## 2. Methods

### 2.1. Population

For this study, MS patients [10] regularly attending the MS Centre of S. Andrea Hospital in Rome were consecutively recruited before starting a first-line disease modifying treatment (DMT) (within a month before starting DMT). Eligibility criteria included women in childbearing age with RRMS aged between 18 and 45 years; an entry score on the Expanded Disability Status Scale (EDSS) [11] ≤5.0 and at least two relapses in the previous 48 months or one relapse during the preceding 12 months; no relapses or steroids intake in the previous 60 days. Exclusion criteria included pathology of the reproductive system; pregnancy or interruption of pregnancy in the previous 12 months; prior immunosuppressive therapy; interferon-beta or any experimental drugs before study entry; glatiramer acetate; and consumption of oral contraceptives in the prior three months. The study protocol was approved by the local ethical committee; each patient provided written informed consent before any study-related procedure.

### 2.2. MRI Acquisition and Analysis

Patients underwent a brain MRI with a 1.5-Tesla Philips scanner. The MRI protocol included proton-density and T2-weighted fast spin-echo sequences, fluid-attenuated inversion recovery sequence, and T1-weighted images obtained before and five minutes after intravenous injection of 0.1 mmol/kg of gadolinium (Gd). Acquisitions were obtained on the axial plane and consisted of 46 contiguous, 3 mm thick slices. MRI images were transferred to the Neuroscience MRI Laboratory of Sapienza University of Rome for analysis. Hyperintense lesion volumes on T2-weighted images and hypointense lesion volumes on T1-weighted postcontrast images were identified and quantified using a semiautomated method (Jim 4.0, Xinapse System, Leicester, UK) to obtain T2 lesion load (T2-LL), T1-hypointense lesion load (T1-LL) calculated on the postcontrast images (i.e., black holes), and Gd-enhancing lesion load (Gd-LL). Volumes of white matter (WM) and grey matter (GM), normalised for subject head size, were estimated with SIENAX [12], part of FSL package [13].

### 2.3. Hormonal Level Dosage

Blood samples were collected during the follicular phase of menstrual cycle. Before the analysis, blood samples were stored in a refrigerator controlled at a stable temperature of −80°C. The chemiluminescence immunoassay method was used to obtain plasma levels of PRL for each patient.

### 2.4. Statistical Analysis

Data are presented as mean (standard deviation) or median (range), as appropriate. Differences between patients with and without gadolinium-enhancing lesion, defined as Active Patients (APs) and Nonactive Patients (NPs), respectively, were assessed with independent samples *t*-test and with Mann-Whitney test as appropriate. Relationship between PRL and cerebral volumes was assessed with partial correlation. To control for the possible influence of age, disease duration, T2-LL, and presence of gadolinium enhancement, on cerebral volumes, these factors were added as correcting variables. Predictors of WM and GM volumes were assessed by means of a multivariate linear regression model in a stepwise fashion (for inclusion: *F* ≥ 1, *p* ≤ 0.005; for exclusion: *F* < 1, *p* > 1). Each model included demographic, clinical, radiological, and hormonal characteristics as covariates. All *p* values less than 0.05 in either direction were considered as significant. Statistical analyses were carried out by a PC version of Statistical Package for Social Sciences, version 17.0 (IBM SPSS, Chicago, IL, USA).

## 3. Results

We included 106 women with a mean (SD) age of 30 (6) years, mean MS duration of 3.3 (3.2) years, and median EDSS of 1.5 (range 0–4.5). Mean PRL level was 12.4 (7) ng/mL, only five patients presented PRL levels above laboratory normal range (5–25 ng/mL) (LNR), and they did not present significant differences in clinical and MRI features compared with the others (data not shown). Sixty-four patients (60%) presented Gd enhancement on MRI.  Table 1 shows demographical and MRI data of patients according to the presence of enhancement on MRI. We found significant lower volume of WM and higher T2-LL and T1-LL in patients with Gd enhancement than in patients without Gd enhancement. No difference was found in PRL plasma levels between the two groups (Table 1). We did not find any correlation between PRL and number of Gd-enhancing lesions or Gd-LL in patients with Gd enhancement even after correcting for age and disease duration (*p* = 0.948 and *p* = 0.734, resp.).

**Figure 1:**
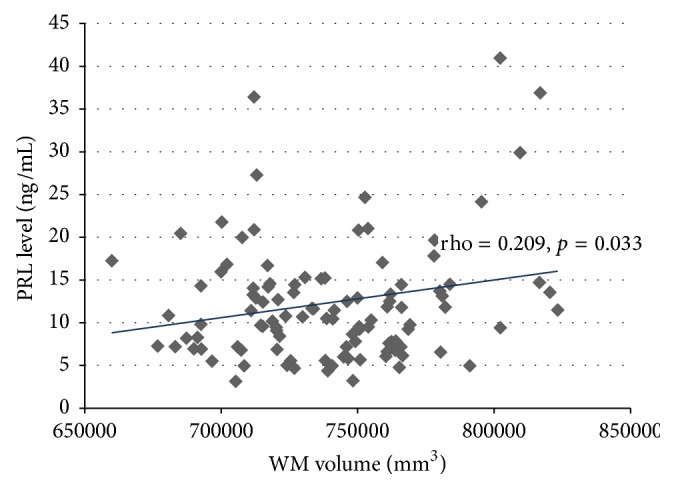
Relationship between PRL plasma levels and WM volume.

Figure 1 shows a significant positive correlation between PRL plasma values and WM volume for the entire study population (rho = 0.209, *p* = 0.033). PRL plasma levels did not correlate with any other clinical or MRI parameters including GW volume (rho = −0.013, *p* = 0.893).

Predictors for WM volume were PRL level (Beta: 1094, *p* = 0.02) and EDSS (Beta: −8742, *p* = 0.015). Predictors for GM volume were T1-LL (Beta: −6.9, *p* < 0.001), age (Beta: −2979, *p* < 0.001), and disease duration (Beta: −2416, *p* = 0.04) (Table 2).

## 4. Discussion

The role of PRL on exacerbating disease or promoting recovery in MS is still debated. Despite the increasing number of studies performed in vitro and on animal models, clinical studies are still poor and include a low number of patients [14]. In this panorama, our study is the first to correlate PRL levels with MRI data from a large cohort of MS patients and adds some relevant piece of information.

First, our data do not support a correlation between PRL and disease subclinical activity: we did not find an increase of PRL levels in patients with active disease as resulting from the presence of Gd enhancement on brain MRI scan. Moshirzadeh et al. reported higher PRL serum levels in patients with MS during relapse than in healthy control supporting the hypothesis that patients with RRMS are in a hyperprolactinemic state [7]. However, differently from Moshirzadeh et al., we studied patients clinically stable; therefore, we could not rule out an increase of PRL during clinical exacerbations as previously reported [7, 8]. Our data seems to be in contrast with even previous report from Yamasaki et al. who showed an increase in serum PRL level during acute relapse involving optic nerve [8]. This apparent discrepancy can be explained by differences in the characteristics of populations included in the two studies: Yamasaki et al. included a high number of patients with elective involvement of optic nerve and spinal cord while we considered only patients showing disseminate central nervous system involvement [8]. Then Berg et al. suggested a role of prolactin in the autoimmune process reporting a relationship between reduction of MRI activity and decline of PRL level after a single intravenous infusion of methylprednisolone [9], while Nociti et al. reported a case in which the relapses were triggered by hyperprolactinemia [15]. Unfortunately, longitudinal data are not available in the present study: a study on PRL fluctuations prior to, during, and after a relapse could clarify if PRL may trigger disease exacerbation rather than reflect an inflammatory status.

Secondly, we showed a positive correlation between white matter volume and PRL in patients suffering from MS. The role of PRL in WM regeneration and repair has been already reported in experimental studies [2, 16, 17] and our data reinforce this idea in clinical setting. Furthermore, PRL acts as protective factor for WM atrophy but not for GM atrophy, suggesting a specific role of PRL in development of WM and in repair of MS damage. This observation is in line with studies indicating a primary involvement of PRL in the regulation of oligodendrocytes production [2, 16]. Gregg et al. demonstrated an increase in oligodendrocytes precursor cells proliferation and oligodendrocytes generation in the maternal CNS and demonstrated that the increased generation of oligodendrocytes during pregnancy is associated with an enhanced capacity to repair demyelination in the maternal central nervous system [16]. A positive effect of PRL on disease course has been also suggested from Zhornitsky et al. [18]. The authors reported that prolactin associated with recombinant murine interferon-*β* reduced severity of EAE compared to PRL alone or to vehicle controls and reduced spinal cord damage in treated mice. Further studies in clinical settings could be useful to study if PRL level is modified during interferon therapy and if this modification is related with clinical response to treatment.

Whether PRL levels are increased in MS patients is still an open question. The lack of a control group of healthy subjects does not allow us to answer this question; however, we did not find a high prevalence of patients with PRL levels above LNR in our population. In a recent review of literature, Zhornitsky et al. [14] suggested that PRL is elevated in some MS patients, likely due to nonspecific dysregulation of the hypothalamic-pituitary-adrenal (HPA) axis, as a result of demyelination. Kira et al. [19] reported the presence of hypothalamic lesions in patients suffering from both MS and hyperprolactinemia: the authors suggested that the increase of serum prolactin may be considered to be a sensitive indicator for hypothalamic lesions in MS. Yamasaki et al. showed that hyperprolactinemia may be one of the characteristic features of patients with MS who preferentially show the optic nerve involvement and discussed the possibility that location of damage could be crucial in the development of hyperprolactinemia associated with MS [8]. More sophisticated MRI studies considering regional damage could help to clarify if the increase of PRL levels reflects an impairment of specific structures rather than progress of inflammation. Furthermore, to better clarify the relationship between PRL and mechanisms of damage and repair, measures of PRL levels in cerebrospinal fluid (CSF) and production of PRL within the CNS should be considered.

Finally a recent case report from Watad et al. [20] raises the question if whatever treats hyperprolactinemia in patients with prolactinoma may prevent the development of autoimmune diseases. We did not observe differences in MRI activity, WM, and GM volumes in patients with PRL levels above LNR compared with the others; however, due to the low number of those patients (5 patients) we cannot exclude a role of PRL in this specific population.

Some important caveats need to be considered in discussing our data. As mentioned above, the major limitation of the study is the lack of a comparison with a population of healthy controls and with a population suffering from a clinical relapse. As another limitation of our study, we could not investigate association of PRL levels with other autoimmune or reproductive system pathologies in MS; in fact, we excluded women suffering from MS in association with other diseases.

Furthermore, the lack of a complete study of the HPA axis limits the interpretation of the results. Overall, our study did not support a role of PRL in subclinical disease activity as detected by MRI, while the positive association of PRL levels with WM volume suggests an involvement of PRL in WM regeneration and repair. Further MRI studies with higher field MRI and more sophisticated analysis of regional damage could help to understand complex role of PRL in MS.

## Figures and Tables

**Table 1:** Demographical and MRI data of patients according to the presence of Gd enhancement on MRI. All values are expressed as median (range) except for age and disease duration that are expressed as mean (SD).

	Patients with Gd enhancement on MRI *N* = 64	Patients without Gd enhancement on MRI *N* = 42	*p*
Age (years)	29 (6)	31 (6)	0.52
Disease duration (years)	2.9 (2.3)	3.6 (3.7)	0.22
Number of relapses in the previous 2 years	1.5 (0–6)	1 (0–3)	0.32
EDSS	1.5 (0–4.5)	1.5 (0–4.5)	0.47
PRL (ng/mL)	11.7 (6.2)	13.4 (8)	0.23
T2-LL (mm^3^)	8516 (59930–1490)	3508 (48675–378)	<0.001
T1-LL (mm^3^)	1458 (15399–45)	477 (15501–0)	0.006
WM volume (mm^3^)	726,750 (659,865–823,248)	750,682 (685,150–820,434)	0.004
GM volume (mm^3^)	810,380 (667344–897,404)	815,054 (694,489–929,6557)	0.231

**Table 2:** Significant predictors of WM volume and GM volume.

Predictors	Beta	95% CI	*p*

White matter volume			
EDSS	−8742	−15727; −1757	0.015
PRL	1094	173; 2014	0.02

Gray matter volume			
Age	−2979	−4205; −1754	<0.001
Disease duration	−2416	−4717; −115	0.04
T1-LL	−6.9	−9.3; 4.5	<0.001
